# Population size, breeding biology and on-land threats of Cape Verde petrel (*Pterodroma feae*) in Fogo Island, Cape Verde

**DOI:** 10.1371/journal.pone.0174803

**Published:** 2017-04-03

**Authors:** Teresa Militão, Herculano Andrade Dinis, Laura Zango, Pascual Calabuig, Laura M. Stefan, Jacob González-Solís

**Affiliations:** 1 Institut de Recerca de la Biodiversitat (IRBio) and Departament de Biologia Evolutiva, Ecologia i Ciències Ambientals, Universitat de Barcelona, Barcelona, Spain; 2 Parque Natural do Fogo, São Filipe, Cape Verde; 3 Centro de recuperación de fauna silvestre de Tafira, Tafira Baja, Spain; Hungarian Academy of Sciences, HUNGARY

## Abstract

Cape Verde petrel (*Pterodroma feae*) is currently considered near threatened, but little is known about its population size, breeding biology and on land threats, jeopardizing its management and conservation. To improve this situation, we captured, marked and recaptured (CMR) birds using mist-nets over 10 years; measured and sexed them; monitored up to 14 burrows, deployed GPS devices on breeders and analyzed activity data of geolocators retrieved from breeders in Fogo (Cape Verde). We set cat traps over the colony and investigated their domestic/feral origin by marking domestic cats from a nearby village with transponders, by deploying GPS devices on domestic cats and by performing stable isotope analyses of fur of the trapped and domestic cats. The population of Fogo was estimated to be 293 birds, including immatures (95% CI: 233–254, CMR modelling). Based on geolocator activity data and nest monitoring we determined the breeding phenology of this species and we found biometric differences between sexes. While monitoring breeding performance, we verified a still ongoing cat predation and human harvesting. Overall, data gathered from trapped cats without transponder, cats GPS trips and the distinct isotopic values between domestic and trapped cats suggest cats visiting the colony are of feral origin. GPS tracks from breeders showed birds left and returned to the colony using the sector NE of the islands, where high level of public lights should be avoided specially during the fledging period. Main threats for the Cape Verde petrel in the remaining breeding islands are currently unknown but likely to be similar to Fogo, calling for an urgent assessment of population trends and the control of main threats in all Cape Verde Islands and uplisting its conservation status.

## Introduction

Seabirds are top marine predators that are suffering a huge decline in their populations over the recent decades due to threats suffered at land, as well as at sea [1,2]. To apply effective conservation measures it is crucial to increase our knowledge about their basic biology and identify their main threats. Unfortunately, this information is often lacking, particularly in secretive species breeding in inaccessible places, such as many gadfly petrel species. Among them, the Cape Verde petrel (*Pterodroma feae*) is a seabird species, breeding in four islands from the Cape Verde archipelago: Santo Antão, Fogo, São Nicolau and Santiago [3]. This species is currently classified as near threatened by the IUCN [4], but this status is poorly supported due to a controversial taxonomy, unreliable population estimates and a lack of knowledge of its breeding biology and main threats, undermining an adequate evaluation of its conservation status and management of the species.

Cape Verde and Desertas petrels have recently been split from the ancestral Zino's petrel (*Pterodroma madeira;* [5,6]) breeding in Madeira Island. A further split between Cape Verde petrel, endemic from Cape Verde, and the Desertas petrels (*Pterodroma deserta)*, breeding in Desertas Is. nearby the Madeira Is., remains more controversial. For example, biometric differences between Cape Verde and Desertas petrel are unclear [7], have not been formally tested and may be obscured by sexual size dimorphism, since no study reported biometric measures of males and females separately. Nevertheless, a growing number of differences, based on bioacoustics [8], molecular genetics [6,9] and spatial ecology [10], support Cape Verde petrel should be considered a different species from its sister taxa. Unfortunately, unlike the Desertas petrel, which has been intensively studied and managed over the last 30 years [11,12], no specific studies and conservation measures have been undertaken on Cape Verde petrels so far.

Concerning population size and breeding biology, most of the previous work on Cape Verde petrel has been occasional and opportunistic and therefore data are scarce and unreliable. A previous estimation of population was of about 500 pairs for all Cape Verde [3], but was based on night calls and therefore rather inaccurate.

Threats on land are of particular concern, since this species is largely exposed to threats arisen from small villages and rural communities settled close to their breeding sites, such as alien invasive mammals, human harvesting and light pollution. As many other seabirds, the Cape Verde petrel evolved in oceanic islands, free of terrestrial predators, and so they lack of effective anti-terrestrial predatory adaptations, making them vulnerable to alien predators [13–15]. For example, cats are responsible for the decline and local extinction of several seabird species of the genus *Pterodroma* [16]. Indeed, a previous study found that Cape Verde petrel was the bird most frequently consumed by feral cats in Fogo Island [17]. Moreover, even though domestic cats receive food from humans, they can also capture and kill wild prey, and can have similar impacts to those of feral cats [18]. Considering the proximity of villages and communities to Cape Verde petrel breeding areas, assessing the domestic or feral origin of cats predating on Cape Verde petrels is crucial to determine adequate management strategies. In addition, Cape Verde petrels were often killed by local people in the past due to the false belief its fat tissue had medicinal benefits. This practice is likely to have severely decimated Cape Verde petrel populations in the past, but its current incidence remains unknown. Light pollution is altering night landscapes worldwide and is increasingly recognized as an important threat for a wide range of organisms vertebrates [19]. Among shearwaters and petrels, fledglings are often disoriented and grounded by artificial light during their first flight to the sea, exposing them to collision and predation, ultimately inducing relevant levels of mortality in several species [20]. This phenomenon has also been reported in gadfly petrels species, for instance in the Barau's (*Pterodroma baraui*) and Hawaiian (*Pterodroma sandwichensis*) petrels, pointing out the sensitivity of this group of birds to this problem [21,22].

Thus, the main aims of our study are: (1) to evaluate if biometry is a useful tool to determine the sex of Cape Verde petrels on the field, i.e., on hand; assess the level of sexual size dimorphism for the species; and provide common biometric measurements for each sex for future comparison purposes with its sister taxa, the Desertas and Zino's petrels; (2) to estimate the population size of Cape Verde petrel in Fogo (Cape Verde); (3) to improve our knowledge on its breeding biology; and (4) to identify the main on-land threats that Cape Verde petrels are exposed to at their breeding grounds.

## Material and methods

### Study area and species

This study was performed inside the Fogo Natural Park, encompassing an area of 8,468.5 Ha located at the top of Fogo Island, with a minimum altitude of 1,000 m [23]. This area holds several endemic species of fauna and flora, a big caldera and the highest point of the Cape Verde (2,829 m), an active volcano which erupted for the last time in November 2014, washing up most of the main village, Chã das Caldeiras, within the caldera.

As all Procellariiform species, Cape Verde petrels only lay one egg per nesting attempt without replacement in case it is lost. Moreover, as many other *Pterodroma* spp. this species breeds in burrows between rocks at more than 800 m of altitude.

### Mark-recapture and sampling at mist-nets

From 2007 to 2016 we mist-netted (without tape luring) Cape Verde petrels at the Bordeira, Fogo Is. during the late incubation period or early chick rearing (February/March) for approximate one week per year. We used 2 mist-nets comprising a total of 18 m with 4 pockets and 20x20mm mesh that were set on the N part of the Bordeira. The mist-nets were opened at the sunset and checked every 5 minutes to minimize the time that birds remained trapped in the mist-net. All birds were ringed and the majority were also measured and sampled for 0.5 ml blood from the tarsal vein. Blood was preserved in absolute ethanol until molecular sexing (methodological details described in S1 Appendix.

### Biometric measurements

For each individual, we measured six biometric variables typically used for seabird species identification (tarsus length, bill length, bill depth at base, bill depth at nostril, maximum head length and compressed wing length). All measurements of Cape Verde petrels included in the present study were taken by the same observer (JGS) using a digital caliper (±0.01 mm), except for wing length, which was measured using a ruler (±0.5 mm). To quantify measurement error, replicate measurements were taken twice on a subset of 19 birds.

To assess biometrics as a potential tool for sexing Cape Verde petrels in the field, i.e., on hand, we first verified if biometric measurements differed between birds of known sex by performing a Student's t-test (function “t.test” of the package “stats” in R 3.3.0 [24]). Subsequently, we randomly selected 80% of females and males to perform a discriminant function analysis (DFA) using “linDA” function from the package “DiscriMiner” [25] in R 3.3.0 [24], assuming equal prior probabilities. The remaining 20% of the birds were used to test the efficiency of the DFA (test-data).

To assess the degree of sexual size dimorphism in Cape Verde petrel, we calculated a sexual dimorphism index following [26]: SSI = ((mean male—mean female)/mean male)*100.

To check for biometric differences among Macaronesian *Pterodroma* species, more specifically between the Cape Verde petrel versus Desertas or Zino's petrel, we performed a Welch modified two-sample t-test with the mean, standard deviation and samples sizes obtained from literature and this study. We performed this analysis using the function "tsum.test" from the package "BSDA" [27] in R 3.3.0[24].

### Population size estimation

For demographic analyses, we selected only birds captured and recaptured in the mist-nets, i.e. we disregarded birds ringed in the nest and those events of birds ringed on mist-net that were recaptured in the nest. Due to the length of the study (10 years) we used the Jolly-Seber open population model with POPAN formulation (Population Analysis http://www.cs.umanitoba.ca/~popan/) in the program Mark (version 6.2, [28]). The POPAN method estimates four demographic parameters: apparent survival probability (ϕ), capture probability (*p*), probability of entrance in the population (*b*), and super-population size (N). The super-population size is defined as the total number of individuals forming part of the population during the study period [29]. The apparent survival, capture and entry probabilities can be time-dependent or constant through time, being represented by the subscripts "t" and "." respectively.

We selected the best model using AICc (Akaike Information Criterion corrected for small sample sizes; [28]). We calculated the super-population size by applying the model averaging approach and its results are presented as mean ± unconditional SE [95% confidence interval]. We also included the percentage of variation attributable to model variation. More information about POPAN method and model selection is described in the S2 Appendix.

To infer which proportion of the population size estimated could correspond to breeding birds, we checked the brood patch stage and assigned a brood patch score to all the captured birds with brood patch information available. The scores used were adapted from [30] to our species: 0 –no brood patch (i.e., no evidence of defeathering); 1 –loss of some down feathers around the edges; 2 –fully developed brood patch, that in this species occurred when almost all the down feathers fall, but there is still a thin anterior-posterior line of down feathers in the middle of the brood patch; 3 –similar to score 2 but there are already sheaths of new down feathers appearing; 4—most of the brood patch area is covered with down feathers that begin to break out of sheaths; 99 –no information available. Examples (photos) of Cape Verde petrels brood patches representative of each score can be found in S1 Fig in supporting information. We compared brood patch scores between breeding birds captured in the nest, with the brood patch of mist-netted birds captured in the same year. As the brood patch stage may vary among individuals, we calculated the number of possible breeders captured on the mist-net in three different ways: (1) a minimum number of possible breeders that only included mist-netted birds with a fully developed brood patch (i.e., the ones with a score of 2); (2) a more probable number of possible breeders that included all the mist-netted birds with a brood patch score 2 and those that have the same brood patch stage as the birds captured in the nest on that year; (3) a maximum number of possible breeders that included all the mist-netted birds except those that we did not had information (score 99) or that do not had any evidence of defeathering (score 0).

### Breeding biology—nest monitoring

From December 2012 to June 2016, we prospected a vast area inside and outside of Fogo Natural Park to locate Cape Verde petrel breeding sites. Every time a nest was found we ringed the adult and its partner whenever possible. In the breeding seasons of 2012/2013 and 2013/2014, nest content was monitored every 3–5 days until chick fledging (except in 2014 around the hatching period when nests were visited every two days to determine the brooding period). In the breeding season of 2014/2015, nest content was monitored every 1–2 days from 21 Feb-14 May, while in 2015/2016 nests were visited trice a month. We monitored the nest by visual inspection with naked eye or by using a Seesnake videoscope (GimateG) when necessary. At the end of the breeding period all accessible juveniles were ringed before fledging.

### Breeding biology—information from geolocators

To determine the date of the first visit to the colony and incubation bouts we used the activity and light data obtained from leg-mounted geolocators-immersion loggers (Mk19, Mk18-H or Mk3005 from BAS or Biotrack) that were deployed to incubating birds and recovered in the following year. Geolocators weighted maximum 2.5g corresponding to 0.9% of bird body mass. Loggers measured light levels every minute and registered the maximum light levels every 5 minutes. Saltwater immersion (wet/dry) data was registered at 3-s intervals using 2 electrodes and, in some models, was stored as the change between the states wet/dry, while in others was stored as the number of positive tests from 0 (continuously dry) to 200 (continuously wet) at the end of each 10-min period. Light and immersion data were used simultaneously to distinguish time spent at sea from time at the colony (darkness in the burrows). Visits of Cape Verde petrels to the colony during the pre-laying period were determined by identifying unusually long dry periods by night (> 4 h, as this was the longest period flying in June when all birds were at sea) and by identifying important light interferences or no light at all during the day (i.e. corresponding to bird remaining in its burrow during the day).

### Predation of Cape Verde petrels

During the breeding seasons of 2012/2013 and 2013/2014, to detect the presence of alien predation (cats *Felis catus* and rats *Rattus rattus*), we checked twice a week for footprints and feces around nest sites and human harvesting on Cape Verde petrels. To check the footprints, we erased any footprint marked in the sand present in front of some nests after checking that them, so all the footprints marked in the sand in the next visit would be new. To confirm and control the occurrence of cats we set 1 in 2013 (from March until June) and 7 in 2014 (from January until June) tomahawk live traps (Tomahawk Live Trap, Hazelhurst, Wisconsin, USA) throughout the breeding area of Cape Verde petrels and checked them twice a week. To assess whether the nearest village (<1 km) to the main breeding area of the Cape Verde petrels, Chã das Caldeiras, could be the source of cats visiting the breeding area, we (1) marked domestic cats of this village with subcutaneous transponder and checked whether cats trapped on the breeding area hold a transponder; (2) studied the movements of 7 domestic cats by deploying GPS tracking devices (CatNip Tech.) programmed to take the position of the animal every 5 minutes (with an accuracy of 15.4±10.1 m; [31]) and; (3) compared the trophic level between domestic cats from Chã das Caldeiras and those trapped at the breeding area, as indicated by carbon (*δ*^13^C) and nitrogen (*δ*^15^N) stable isotopes analyses of fur samples. Cat tracks were mapped using ArcGIS 10 [32].

Fur samples were prepared and analyzed for stable isotopes analysis as described in S3 Appendix and isotopic values of standard material used is presented in S1 Table. Normality of the isotopic values was checked using Shapiro-Wilk’s test (all p>0.117) and feral and domestic cat values compared using Student's t-test. All statistical analyses were performed with PASW Statistics 18 [33] assuming a critical p-value of 0.05.

### Light pollution

Light-induced mortality is known to occur in other gadfly petrels, especially in fledglings [21,22], pointing out the possibility to also occur in Cape Verde petrels. To identify where conservation actions should be applied in the future to avoid high light pollution levels, and thus reduce the probability of light-induced mortality in the study species, we deployed 15 GPS devices on 9 breeding adults over the chick-rearing period of 2015. We assumed that routes taken by breeding adults from the breeding colony to the sea and vice versa would be similar to the ones of the fledglings when they leave the nest in direction to the sea, identifying the main area where light pollution levels should be maintained low. We recovered 10 of GPS deployed and we were able to download the data from 6 of them, although only 5 had positions on land. GPS were encased in a shrinking tube to avoid contact with water and deployed on the back of the birds, attached to the mantel feathers using Tesa tape. GPS devices model was GiPSy-4, with a little size (23 x 15 x 6 mm) and weighed about 10g (TechnoSmArt). Birds normally weighted around 300g, and therefore the GPS represents 3.4% of the body mass. All GPS devices were configured to take a position every 15 minutes. To minimize the impact of GPS deployments, we always alternated them between partners of the same nests. When a GPS tracked bird returned to the nest, we removed the GPS of this bird and deployed a new one to the partner. In 2015 during the GPS fieldwork, the nests were monitored every day and it was easy to check the back of the birds by visual inspection with naked eye or by using a Seesnake videoscope (GimateG) when necessary. Light pollution levels of Fogo Island were obtained from a cloud-free composite of VIIRS (Visible Infrared Imaging Radiometer Suite) night-time lights corresponding to the last year available (2013) Stable Light Product from Defense Meteorological Satellite Program—Operational Linescan System (DMSP-OLS) Nighttime Lights Time series (Version 4). Stable Light Products contain the lights from cities, towns, and other sites with persistent lightning, whereas ephemeral events such as fires are discarded (National Oceanic and Atmospheric Administration (NOAA) National Geophysical Data Center, available at: https://www.ngdc.noaa.gov/eog/dmsp/downloadV4composites.html). Urban areas of the island were obtained from Natural Park Management Plan of Fogo [23] and mapped using ArcGIS 10 [32].

All values are indicated as means ± standard deviation, unless otherwise indicated.

### Ethic statement

All petrels and cats were handled in strict accordance with good animal practice as defined by the current European legislation. The deployment of geolocators in Cape Verde petrels did not take more than 10 minutes and, on no occasion, it had visible deleterious effects on study animals. The deployment of transponders in cats was performed by an experienced veterinary (Pascual Calabuig) and the fur was carefully cut. All the procedures to domestic cats (sterilization, transponder and GPS deployment) were performed with the agreement of their owners. All work was approved by Direção Geral do Ambiente (now called Direção Nacional do Ambiente, DNA) of Cape Verde (research permits 01/2009, 02/2010, 01/2011, 01/2012, 04/2013, 018/2014). The DNA only began to emit research permits in 2009.

## Results

### Molecular sexing and biometric measurements

We determined the sex of 141 mist-netted adults (61 males and 80 females) but 4 mist-netted birds could not be blood-sampled and therefore could not be sexed molecularly. The molecular sex of 23 birds was determined twice and sexing was always consistent.

We took biometric measurements from 121 Cape Verde petrels captured in mist-nets (Table 1): 49 males and 72 females. To quantify measurement error, replicate measurements were taken twice on a subset of 19 birds. Intra-class correlations ranged from 0.612 (p = 0.002) for the bill depth at nostril to 0.964 (p<0.001) for the maximum head length. Cape Verde petrels showed a sexual size dimorphism index of 0.6–4.4% (depending on the biometric measurements, Table 1), with females being significant smaller than males in all measurements except in the bill length (tarsus length: t = 2.823, d.f. = 119, p = 0.006; bill length: t = 1.108, d.f. = 119, p = 0.270; bill depth at base: t = 5.634, d.f. = 119, p<0.001; bill depth at nostril: t = 6.554, d.f. = 119, p<0.001; maximum head length: t = 5.669, d.f. = 119, p<0.001; wing: t = 3.058, d.f. = 119, p = 0.003) after the Bonferroni correction (critical p-value of 0.008).

**Table 1:** Biometric measurements (mm) and dimorphism index values (SDI, %) of Cape Verde, Desertas and Zino's petrels.

Species	Sex	n	Tarsus length	n	Bill length	n	Bill depth at base	n	Bill depth at nostril	n	Maximum head length	n	Compressed wing length	Source
**Cape Verde petrel (*Pterodroma feae*)**	Males	49	35.27±1.00	49	29.05±0.75	49	13.91±0.46	49	9.85±0.39	49	73.59±1.16	49	274.6±4.6	This study
	Females	72	34.76±0.96	72	28.88±0.84	72	13.47±0.40	72	9.42±0.33	72	72.26±1.34	72	271.8±5.3	This study
	Both	121	34.97±1.00 (SDI = 1.4%)	121	28.95±0.81 (SDI = 0.6%)	121	13.64±0.48 (SDI = 3.2%)	121	9.59±0.41 (SDI = 4.4%)	121	72.80±1.43 (SDI = 1.8%)	121	273.0±5.2 (SDI = 1.0%)	This study
**Desertas petrel (*Pterodroma deserta*)**	Both	185	38.0±1.6	258	29.7±1.1	306	14.7±0.7	256	10.4±0.6			337	271.0±5.9	[5]
**Zino's petrel (*Pterodroma madeira*)**	Both	62	34.3±1.3	71	25.8±0.9	71	11.3±0.5	71	8.0±0.4			70	251.1±4.8	[5]

Mean and standard deviation (SD) of the biometric measurements (mm) of Cape Verde petrel capture in mist-nets in Fogo Island (Cape Verde) in 2007–2016, Desertas petrel breeding in Desertas Islands and Zino's petrel breeding in Madeira Island. For Cape Verde petrel, sexual size dimorphism index values (SDI, %) are showed between parenthesis.

From the 121 adults of known sex, we used the biometric measurements 39 males and 58 females, randomly selected as training data to constructed a discriminant function analysis (DFA), and the remaining 10 males and 14 females were used to test the DFA obtained (test data). The training and test data achieved 77.3% and 91.7% of correct classification of the birds, respectively, so a total of 97 out of 121 feathers were correctly classified (80.2%). For this DFA, we obtained the following discriminant function coefficients:

D [males] = 13.79*(tarsus length) + 16.55*(bill length) + 26.23*(bill depth at base) -16.02*(bill depth at nostril) + 29.57*(maximum head length) + 8.31*(wing length)– 2791.11
D [females] = 13.43*(tarsus length) + 17.77*(bill length) + 24.83*(bill depth at base) -17.62*(bill depth at nostril) + 28.64*(maximum head length) + 8.14*(wing length)– 2712.42

With these function, adult birds with D[males]>D[females] are males; if the opposite occurs they are females.

We compared our biometric measures of Cape Verde petrels with those available in literature for the rest of Macaronesian petrels (see Table 1). Considering a critical p-value of 0.010 after the Bonferroni correction, Cape Verde petrels had significantly smaller biometric measures (tarsus length: t = -20.381, d.f. = 303.45, p<0.001; bill length: t = -7.458, d.f. = 309.31, p<0.001; bill depth at base: t = -17.903, d.f. = 318.17, p<0.001; bill depth at nostril: t = -15.320, d.f. = 327.82, p<0.001) than those from its sister taxon breeding in Desertas Islands, except for wing length (t = 3.499, d.f. = 238.38, p = 0.001), which was longer in Cape Verde petrel. On the contrary, Zino's petrels showed significantly smaller biometric measures than Cape Verde petrels (tarsus length: t = 3.555, d.f. = 98.98, p = 0.001; bill length: t = 24.281, d.f. = 134.61, p<0.001; bill depth at base: t = 31.769, d.f. = 141.96, p<0.001; bill depth at nostril: t = 26.344, d.f. = 149.72, p<0.001; wing length: t = 29.460, d.f. = 153.75, p<0.001).

### Population size estimation

From 2007 to 2016 we captured 145 Cape Verde petrels and recaptured 77 birds (55 different individuals) (Table 2). The goodness-of-fit of the CJS model was not significant for global test (X^2^ = 26.293, d.f. = 29, p = 0.610), neither for the Test2 (Test2.CT X^2^ = 0.729, d.f. = 7, p = 0.998; Test2.CL X^2^ = 5.936, d.f. = 6, p = 0.430) and Test3 (Test3.SR X^2^ = 13.034, d.f. = 8, p = 0.111; Test3.SM X^2^ = 6.595, d.f. = 8, p = 0.581). So, the CJS model fit the data well and there was no evidence of transients or trap-dependence.

**Table 2:** Reduced m-array summary of the capture-mark-recapture data set of the Cape Verde petrels captured and recaptured with mist-nets inside the Fogo Natural Park, Cape Verde, from 2007–2016.

Occasion	Released	Time of first recapture									Total birds recaptured
		2008	2009	2010	2011	2012	2013	2014	2015	2016	
2007	20	1	1	6	0	0	0	0	0	0	8
2008	18		2	4	3	0	2	0	0	0	11
2009	19			7	1	2	0	0	0	0	10
2010	33				4	4	0	2	0	0	10
2011	15					4	0	0	1	0	5
2012	32						5	9	0	4	18
2013	14							3	1	0	4
2014	29								2	2	4
2015	9									3	3

The column "released" includes the number of birds released per year, i.e., the birds captured for the first time plus the birds recaptured in that year. The columns of "time of first recapture" included the number of birds per year that were recaptured for the first time during the study period. The majority of Cape Verde petrels were recaptured for the first time in the first three years after being marked.

The most parsimonious model, i.e., the one with the lowest AICc, was the model 1 ϕ(.)*p*(t)*b*(.) (Table 3), in which the apparent survival and probability of entrance were constant and the capture probability was time-dependent. This model estimated an apparent survival probability of 0.76±0.04 (SE) [95% CI: 0.67–0.83], a probability of entrance in the population of 0.07±0.01 (SE) [95% CI: 0.05–0.10] and capture probabilities ranging from 0.11±0.04 (SE) [95% CI: 0.05–0.23] to 0.40±0.08 (SE) [95% CI: 0.24–0.54]. Moreover, this model reached AICc weight of 0.78356, which means that comprises 78.4% of support in the data, followed by the model ϕ(t)*p*(t)*b*(.) with a AICc weight of 0.20255 and a ΔAICc of near 3 (Table 3). The rest of models showed a ΔAICc >4 in relation to the most parsimonious model (Table 3).

**Table 3:** Capture–mark–recapture models performed to estimate the population size of the Cape Verde petrels inside the Fogo Natural Park, Cape Verde.

Model number	Model design	AICc	ΔAICc	AICc weight	N° of parameter
**1**	Φ(.) *p*(t) *b*(.) N	490.7717	0	0.78356	13
**2**	Φ(t) *p*(t) *b*(.) N	493.4774	2.7057	0.20255	20
**3**	Φ(t) *p*(.) *b*(.) N	500.3783	9.6066	0.00643	12
**4**	Φ(.) *p*(t) *b*(t) N	500.4296	9.6579	0.00626	20
**5**	Φ(t) *p*(.) *b*(t) N	505.7537	14.9820	0.00044	20
**6**	Φ(.) *p*(.) *b*(.) N	506.3919	15.5702	0.00033	4
**7**	Φ(t) *p*(t) *b*(t) N	506.3672	15.6255	0.00032	27
**8**	Φ(.) *p*(.) *b*(t) N	508.4389	17.6672	0.00011	12

Models are ranked according to AICc values, with model 1 being the most parsimonious model, i.e., the model which best explains the variation in the data while using the fewest parameters. ΔAICc corresponds to the difference between the AICc of each model and the AICc of model 1. AICc weight is the normalized Akaike weight which can be interpreted as the proportion support in the data for a given model. The deviance of the models is not presented here because they cannot be calculated in Mark for this kind of models. The symbol Φ, *p* and *b* represent respectively the apparent survival, the capture probability and the probability of entrance in the population. The symbol "t" and "." stand for time-dependent and constant through time, respectively.

Based on the model averaging, the super population size was estimated on 293 birds ± 31 (unconditional SE) [95% CI: 232–354] and the percentage of variation attributable to model variation was of 0.71%. Based on the comparison between the brood patch scores of the birds captured in the nests and in the mist-net, 40% of the birds captured in the mist-net could be breeders, corresponding to ~117 [95%IC 89–142] breeding birds (Table 4). However, as the brood patch scores can vary among individuals, we also calculated a minimum and a maximum number of possible breeders. Thus, the number of possible breeders of our estimation could range from 49 [95%IC 39–59] to 199 [95%IC 157–240] birds.

**Table 4:** Brood patch scores of Cape Verde petrels captured in nest and in mist-net and the possible number of breeders among mist-netted birds.

Year	Date	Capture location	Brood patch score						Total	Number of possible breeders		
			99	0	1	2	3	4		Min (%)	Most probable (%)	Max (%)
**2011**	05 Mar	Nest	0	0	0	0	1	0	1	-	-	-
**2012**	12–17 Feb	Nest	0	0	0	7	0	0	7	-	-	-
**2013**	07–11 Mar	Nest	1	0	0	1	1	2	5	-	-	-
**2014**	18–28 Feb	Nest	3	0	0	3	3	0	9	-	-	-
**2015**	21 Feb—07 Mar	Nest	7	0	0	6	4	0	17	-	-	-
**2016**	03–06 Mar	Nest	2	0	0	0	1	0	3	-	-	-
**Total**			**13**	**0**	**0**	**17**	**10**	**2**	**42**			
**2007**	17–21 Mar	Mist-net	8	1	0	0	1	10	20	-	-	-
**2008**	05–09 Mar	Mist-net	0	0	0	0	1	17	18	-	-	-
**2009**	18–21 Feb	Mist-net	5	0	0	0	0	14	19	-	-	-
**2010**	01–04 Feb	Mist-net	0	3	16	0	12	2	33	-	-	-
**2011**	05–08 Mar	Mist-net	0	6	0	0	7	2	15	0 (0%)	7 (46.7%)	9 (60.0%)
**2012**	12–17 Feb	Mist-net	1	8	7	12	4	0	32	12 (37.5%)	12 (37.5%)	23 (71.9%)
**2013**	05–11 Mar	Mist-net	0	5	0	1	4	4	14	1 (71%)	9 (64.3%)	9 (64.3%)
**2014**	19–24 Feb	Mist-net	0	7	12	7	3	1	30	7 (23.3%)	10 (33.3%)	23 (76.7%)
**2015**	19–23 Feb 13 Mar	Mist-net	0	4	2	2	1	0	9	2 (22.2%)	3 (33.3%)	5 (55.6%)
**2016**	02–06 Mar	Mist-net	0	6	13	3	4	2	28	3 (10.7%)	7 (25%)	22 (78.6%)
**Total**			**16**	**46**	**51**	**25**	**37**	**52**	**227**	**Mean = 4.2 (16.8%)**	**Mean = 8.0 (40.0%)**	**Mean = 15.2 (67.8%)**

The scores used were adapted from [30] to our species: 0 –no brood patch (i.e., no evidence of defeathering); 1 –loss of some down feathers around the edges; 2 –fully developed brood patch, that in this species occurred when almost all the down feathers fall, but there is still a thin anterior-posterior line of down feathers in the middle of the brood patch; 3 –similar to score 2 but there are already sheaths of new down feathers appearing; 4—most of the brood patch area is covered with down feathers that begin to break out of sheaths; 99 –no information available. As the brood patch stage may vary among individuals, we calculated the number of possible breeders captured on the mist-net in three different ways: (1) a minimum number of possible breeders that only included mist-netted birds with a fully developed brood patch (i.e, the ones with a score of 2); (2) a more probable number of possible breeders that included all the mist-netted birds with a brood patch score 2 and those that have the same brood patch stage as the birds captured in the nest on that year; (3) a maximum number of possible breeders that included all the mist-netted birds except those that we did not had information (score 99) or that do not had any evidence of defeathering (score 0).

### Breeding biology—nest monitoring and information from geolocators

From December 2012 to June 2016, we identified 14 borrows as potential nest sites visited by Cape Verde petrels during the pre-laying period (Table 5). The majority of the burrows (93%) were located inside the Fogo Natural Park, but one of them (burrow 7) was located in Monte Preto, in the buffer area of the Park. Moreover, we found some evidences (footprints and droppings) of the presence of Cape Verde petrel in other areas of the Fogo Natural Park, near Achada Grande (Mosteiros) and Fernão Gomes (Monte Velha).

**Table 5:** Description of the activity detected in the burrows monitored during four breeding seasons.

Burrows	Breeding season 2012/2013	Breeding season 2013/2014	Breeding season 2014/2015	Breeding season 2015/2016
**1**	Successful	Successful	Successful*	Successful
**2**	Adult killed while incubating	Empty	Empty	Empty
**3**	Successful	Successful	Successful*	Successful
**4**	No egg	Adult predated by a cat	Empty	Empty
**5**	Successful	Successful	Chick disappeared	Successful
**6**	Active nest but birds inaccessible	Active nest but birds inaccessible	Broken egg	Egg abandoned
**7** **(located outside the Fogo Natural Park)**	Egg disappeared	Reproduction not confirmed	Empty	Empty
**8**	No egg	Empty	Empty	Empty
**9**	Successful	Successful	Egg abandoned*	Empty
**10**	Successful	No egg	Successful*	Successful
**11**	-	Reproduction not confirmed	Dead chick*	Successful
**12**	-	Successful	Successful*	Successful
**13**	-	-	No egg but adult with brood patch developed	Empty
**14**	-	-	-	No egg
**Number of confirmed active nests**	7	5	8	7
**Number of fledglings**	5	5	4	6
**Annual breeding success**	71%	100%	50%	86%

We considered that a breeding pair was: "successful" when there was juvenile in the nest during the fledging period; "No egg" when an adult was found in the burrow but without egg; and "empty" when neither an egg nor an adult was found in the nest. In the burrow 6, we were able to see an adult in the nest during the incubation period but it was inaccessible, so the breeding success could not be confirmed. "Reproduction not confirmed" occurred when there were some feces and footprints during all the breeding period, but we did not find any egg or adult bird.

"-" means that the content of that nest was unknown for that breeding season.

"*" means that GPS devices were deployed to at least one of the breeding adults of that burrow.

From the 14 burrows, two of them (the burrows 8 and 14) were never occupied during any of breeding seasons and thus not considered as active nests. In the first two breeding period, burrow 6 was occupied by day during the incubation period but the nest site was quite inaccessible and out of sight, consequently we could not confirm its breeding success. Afterwards, we found a new entrance to that nest and the breeding success could be confirmed. In 2012/2013, the adult incubating in the burrow 2 was harvested by local teenager (this event was confirmed by his father). Moreover, in 2012/2013, the egg and adult from the burrow 7 disappeared with no traces of egg shells and in 2014/2015 the chick of the burrow 5 disappeared with no traces of a cadavers and before the fledging period, suggesting these were also harvested by locals. The number of chicks that fledged successfully per year varied between 4 and 6 (Table 5). Thus, taken into account only the accessible burrows which we could confirm the presence of an egg, the breeding success varied between 50% and 100% (Table 5).

Regarding the breeding phenology, in December we detected several evidences that Cape Verde petrels were visiting the colony at night, as indicated by footprints and droppings inside and outside the borrows. However, activity data from geolocators showed that birds started visiting breeding areas on average on 24^th^ September (±12 days, N = 7; S2 Table). Females laid their egg from 15 January to 8 of February (N = 5 nests). The incubation period lasted on average 50 days (±0.6 days, N = 5) and both partners shared the incubation duties, performing incubation bouts of 12 days (±5 days). Chicks hatched from 26 February to 1April (N = 4 nests) and the chick rearing period lasted on average 92 days (±11 days, N = 4). The brooding period lasted up 4 days (N = 2 nests). After that period, adults visited the burrow only by night to feed their chicks, although activity data from 7 geolocators of 5 adults revealed 2 of them occasionally remained during daylight for one day after the brooding period. All chicks fledged before 14 June (N = 5). Overall, 95% of birds captured in a burrow in one year (N = 20 birds) were recaptured in the following years in the same burrow. Only one bird was found during 2 years breeding in one nest and the following 2 years was found near or within another nest but without an egg.

### Predation on Cape Verde petrels and origin of trapped cats

**Fig 1:**
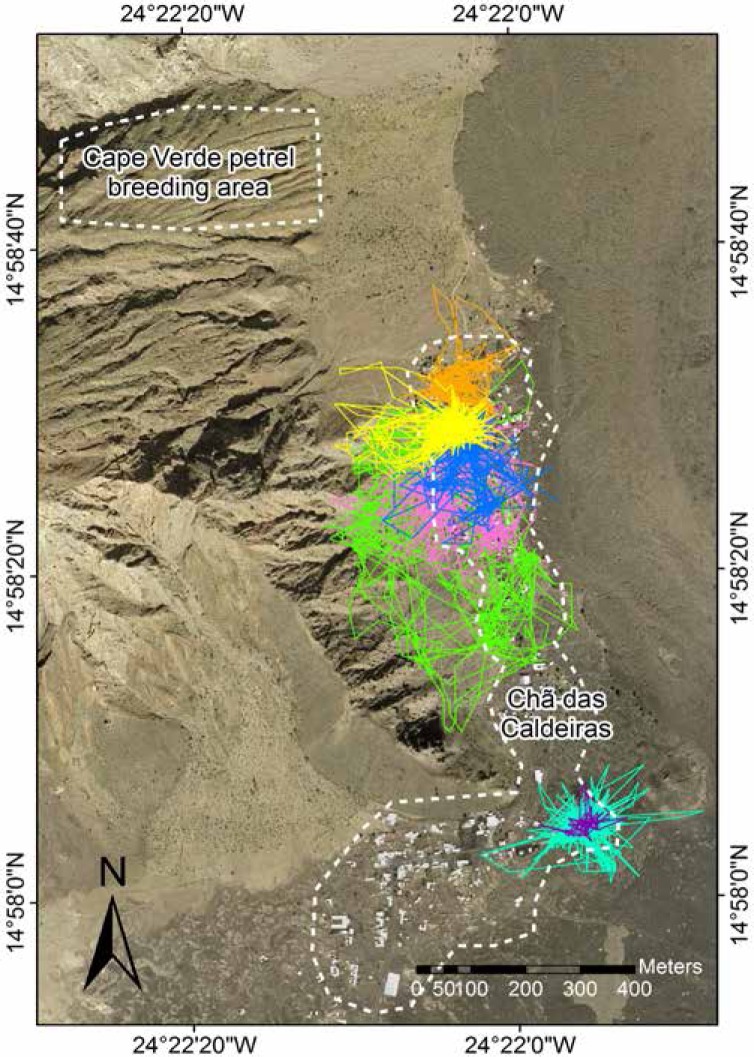
GPS tracks of the seven domestic cats from Chã das Caldeiras. Each cat track is depicted in a different color. Note that none of the cat tracks overlaps with the breeding area. Map courtesy of Instituto Nacional de Gestão de Território (Cape Verde).

Beside the birds harvested by local people commented above, we confirmed the presence of rats (*Rattus rattus*) in the breeding area as we found two specimens dead near the nests. We also found several cat scats and footprints in the main breeding area as well as Cape Verde petrel remains, indicating cats predated at least three adults (one in 2012/2013 and the other 2 in 2013/2014). Moreover, within the breeding area we captured 1 and 7 cats in 2013 and 2014, respectively. In 2013 we identified 32 domestic cats in Chã das Caldeiras, which 25 were marked with a subcutaneous transponder 2013 (previously to the deployment of the cat traps) and 7 in 2014. None of the cats trapped at the breeding area wore a transponder. In 2014, we deployed and recovered 7 GPS devices on domestic cats from Chã das Caldeiras with tracks of 7 to 13 days. None of the tracked cats visited or even approached the breeding area (Fig 1), although one of them (green track in Fig 1) visited some areas clearly out of the village. All the detail information of GPS positions of each tracked cat is available in S3 Table.

**Fig 2:**
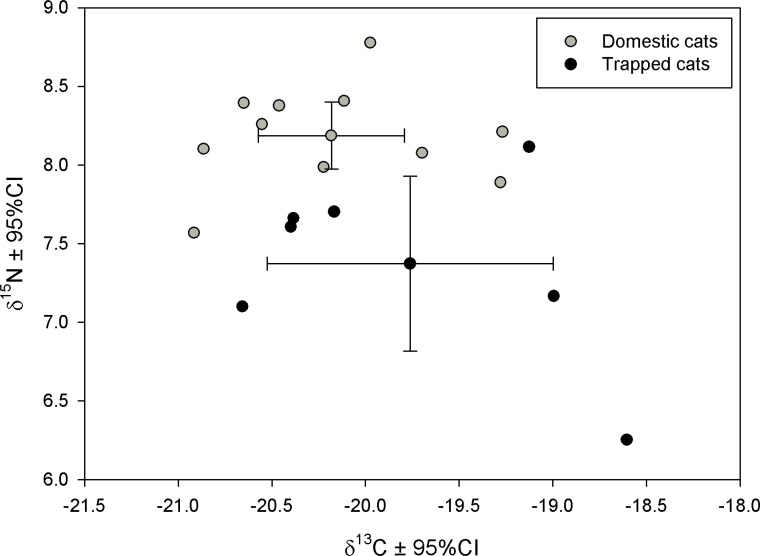
Fur stable isotopic values of domestic cats and those trapped at the Cape Verde petrel colony. Fur stable isotopic values of domestic cats of Chã das Caldeiras (grey dots) and from cats trapped at the Cape Verde petrel breeding area (black dots). Each point represents the isotopic value of a single cat and crosses indicate the mean ± 95% confidence interval for each group of cats. Trapped cats have in general lower δ^15^Nvalues than domestic ones.

Regarding to the stable isotope analysis in fur, *δ*^15^N values of domestic cats from Chã das Caldeiras were significantly higher than those from cats trapped at the breeding area (*δ*^15^N 8.19±0.32SD and 7.37±0.60SD, respectively; t = 3.775, d.f. = 16; p = 0.002; Fig 2), whereas we did not find significant differences between domestic and trapped cats in *δ*^13^C (*δ*^13^C -20.18±0.58SD and -19.76±0.83SD, respectively; t = 1.272, d.f. = 16; p = 0.222).

### Light pollution

**Fig 3:**
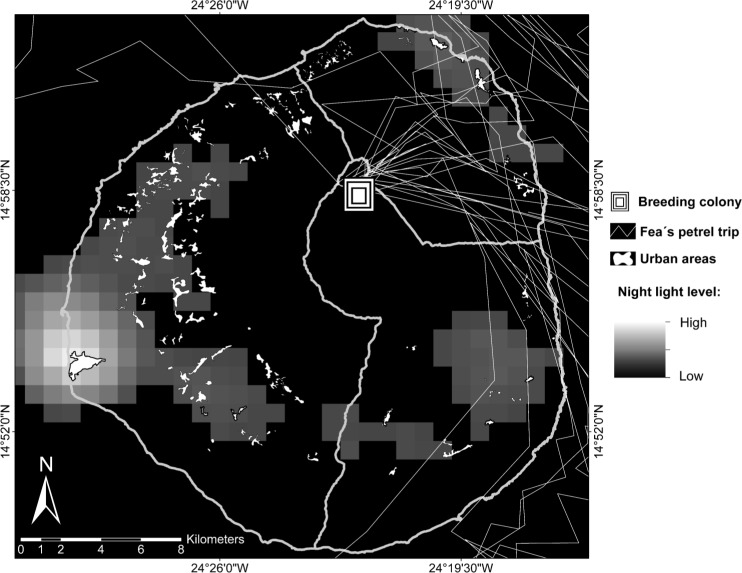
Departure and return trajectories of Cape Verde petrels from the colony in Fogo Island (Cape Verde). GPS trajectories of Cape Verde petrels leaving and returning from the colony during the chick-rearing period. Light pollution gradient was taken from a cloud-free composite of VIIRS night time lights corresponding to 2013 (data from NOAA National Centers for Environmental Information). The location of urban areas of the island was obtained from the geographic database Fogo National Park and the thicker lines represent the municipal limits.

GPS tracks showed that the majority of the breeding birds went in and out of the breeding colony through the north-east sector of the island (Fig 3). All the detail information of GPS positions of each tracked Cape Verde petrel is available in S4 Table.

## Discussion

In this study, we found biometric differences in size in Cape Verde petrels, we confirmed its reproduction inside and outside the borders of the Fogo Natural Park and its small population size. Additionally, we also showed that two of the major threats suffered in the past by this species are still present today, with Cape Verde petrels being killed by local people and predated by cats. Finally, we revealed in which area light pollution levels should be maintained low during the Cape Verde petrel breeding period to avoid light-induced mortality of these petrels.

Sexual dimorphism found in this study concur with that found in other Procellariidae species [26,34,35], with higher values of sexual dimorphism index for bill depth measurements than for any other measurement. Cape Verde petrel females were 0.6 to 4.4% smaller than males (depending on the biometric measurements), however these differences between sexes only allowed to correctly identify the sex of 80.2% of the birds. Regarding the biometric comparison between Macaronesian petrels, overall Cape Verde petrels from Fogo Island showed intermediate sizes between Zino's and Desertas petrels. The only exception was the wing length, which was greater in Cape Verde petrels, however this could result from methodological differences in the wing length measurement (flattened and stretched in our study). Size differences among the 3 species could be an artefact as each species was measured by a different person. Nevertheless, our biometric results are in line with previous studies [7], suggesting significant biometric differences could really exist among the three taxa. So far biometric comparisons among taxa did not consider the sex of the birds. We have shown that sex ratio of captured birds may depart from parity (49 males versus 72 females with biometric data) and males and females of Cape Verde petrels differ significantly in their biometry. Therefore, biometric differences among taxa previously reported may be biased or obscured not only by differences among measurers but also by the gender composition of the sampled birds. Size differences or lack of them among the three taxa should be taken with caution and further confirmed by a single person measuring sexed birds from the three species.

We provide the first reliable estimate of Cape Verde petrel population size in Fogo in ca. 293 birds, based on capture-mark-recapture from mist-nets. However, some lines of evidence suggest this estimation includes not only breeders, but also immature birds from different ages. Four of the 12 chicks born since 2012 were recaptured at the mist-nets 2 or 3 years after being ringed, evidencing that we are capturing young immatures in the mist-net. Additionally, two mist-netted birds captured in 2007 were deployed with geolocators which were recovered in 2010, when these birds were recaptured again at mist-net. These birds did not show any signs of incubation bouts during those years, suggesting these birds did not breed during three years. These evidences indicate birds caught at mist-nets are mainly immature and non-breeders visiting the area for displaying and courting. Probably, once birds are about 5 years old and find a partner and start breeding, they display and visit the courting area to a lesser extent. The fact that we are capturing all these immature birds would explain the relatively low survival probability (0.76) compared to mean adult survival estimates of Procellariform species (0.93, [36]), since immature birds have lower survival rates. Indeed, with this low adult survival probability, Cape Verde population within the Fogo Natural Park would tend to decrease until extinction. Based on the brood patch score of the mist-netted birds, 40% of birds captured were possible breeders, but of the 20 birds breeding in the known nests only one was recaptured in the mist-net. If most Cape Verde petrels breeding within the Fogo Natural Park are not being captured because they do not overfly the area where the mist-net are set, the survival and demographic parameters may be underestimate. Nevertheless, the number of possible breeders captured in mist-net is indicating that we are capturing breeders from other breeding areas still not found, suggesting that our population size estimation includes breeders from different breeding areas. Breeders from other colonies located far from the mist-net area may not be captured. However, based on a previous study, the largest colony of Cape Verde petrel is located in Chã das Caldeiras [3], so we believe our estimation although with some limitations previously explained would be representative of the population size of Cape Verde petrels in Fogo. Based on the nests known and on the estimation of 117 possible breeders captured in the mist-net, the number of breeding pairs within the Park would probably not overcome 100 pairs, which would agree with the 80 pairs previously estimated by [3]. Nevertheless, the number of breeding pairs is difficult to estimate, as there can be other breeders that may not be captured in the mist-net. We suggest that future studies should deploy small GPS/GSM in breeders captured at the mist-net and perform intensive nocturnal calling survey to find new breeding areas to improve the estimation of breeding pairs.

Major differences among the Cape Verde population and those from the rest of Macaronesian gadfly petrels can be found in the timing of breeding. The phenology of Cape Verde petrels inferred from burrow monitoring and activity from geolocators generally matched with the breeding period known for this taxon, i.e. from November to May [7]. However, we found that some chicks can fledge in June and geolocator data revealed some birds may start visiting the breeding area at night as early as end of August. During most of this period, Desertas and Zino’s petrels are wintering and therefore they have little chance to interbreed with those from Cape Verde. This asynchrony in the breeding phenology can contribute to speciation in another Procellariiform species [37] and thus supports a currently independent evolution of the three lineages [6].

Taking into account the small population size estimated for Fogo, it is crucial to identify their main threats. We confirmed that at least one adult was killed by local teenager (and maybe another adult and a chick), and we detected the presence of rats and cats within the Cape Verde petrel breeding area. Indeed, at least three adults were predated by cats, agreeing with a previous study that found Cape Verde petrel remain in cat scats in Fogo near the breeding area of our study species [17]. Predation by feral cats occurs in several island worldwide and these predators are responsible for several seabird population declines and local extinctions [16]. In addition, domestic cats can also have important population level impacts on seabird populations [18]. Therefore, it is crucial to assess the feral or domestic origin of cats predating on seabirds, since in the first case the control of free-ranging domestic cats should include all villages from the island whereas in the latter case should focus on villages nearby the breeding areas. In Chã das Caldeiras (at less than 1 km from the main breeding area of Cape Verde petrels), domestic cats are mainly free-ranging but GPS tracking showed cats moved within a fairly small area centered in the house of their owner (generally <200 m), in line with previous studies [38,39]. Most importantly, tracked cats did not approach the breeding area of Cape Verde petrels and none of the cats trapped at the colony carried a transponder. In addition, stable isotope analyses on cat fur indicated that the trophic level of cats from Chã das Caldeiras was greater (higher δ^15^N values) than that of trapped cats on the breeding area. Feral cats from Fogo feed on small mammals and a wider range of prey of low trophic levels, such as, insects, reptiles and birds [17,40], which possibly show lower δ^15^N values compared to the diet of domestic cats. Domestic cats feed on the same prey as feral cats, but they are also regularly fed by their owners on canned food or food remains (e.g. meat and bones from poultry, goats and fish, etc.) with high δ^15^N values than their natural prey. Taken together, all results suggest cats trapped in the breeding colony are from feral origin, indicating efforts on cat control should primarily focus on feral cats wandering in and around the Cape Verde petrel breeding areas and on sources of feral cats across the island. These measures should ideally be accompanied by a parallel rat control to avoid an increase in rat population, which may ultimately have more impact on Cape Verde petrel than cat predation [13].

Light-induced mortality occurs in several gadfly petrels, especially in fledglings [21,22], suggesting that Cape Verde petrels could also be sensitive to light pollution. Indeed, fledglings have been collected in Chã das Caldeiras near light sources in at least three occasions over the last decade (Fogo Natural Park authorities’ personal communication), despite no public lights or infrastructures with important sources of light in the village. In our study, GPS trajectories of breeders showed birds preferred to cross areas at NE of the island in their nocturnal trips to and from the sea. If fledglings use the same routes as adults to reach the sea, an increase in light pollution in the NE of Fogo island could ultimately increment the number of grounded fledglings of the study species. More detailed studies are needed to assess if Cape Verde petrel trajectories may also be influenced by the topography of the islands and/or by prevailing winds and if they change trajectories among years. Nevertheless, we call for a control of public light and other sources of nocturnal light in NE the island.

## Conclusions

Cape Verde petrels showed an intermediary size and a distinct breeding phenology than Desertas and Zino’s petrels. Within Cape Verde, the population of Fogo is among the best preserved and the only one that has been systematically studied over the last 10 years so far. Its small population size and the proximity of its main breeding area to local villages makes this population particularly vulnerable to threats on land, calling for a careful re-evaluation of its conservation status. In fact, main threats detected in the present study are directly related to the proximity of villages and small communities and thus likely to be similar across all main breeding sites in Cape Verde, which are all located in inhabited islands. Therefore, it is crucial to expand monitoring and control of main threats to the other three islands in Cape Verde known to hold breeding populations of Cape Verde petrels, i.e. monitoring of population dynamics and trends, locating new breeding areas, re-evaluating limits of protected areas in order to include the entire breeding grounds of the Cape Verde petrel, controlling human harvesting and cat and rat predation, controlling light pollution and increasing awareness of the Cape Verde petrel threats among local communities and villages. However, all threats addressed in the present study mainly refer to threats on-land, but Cape Verde petrel may also suffer threats at sea, and therefore the present assessment should be extended to the marine environment in the near future.

## Supporting information

S1 Appendix Description of molecular sexing methodology. (DOCX)

S2 Appendix Description of POPAN Jolly Seber modelling and model selection. (DOCX)

S3 Appendix Description of the stable isotope analysis of fur samples. (DOCX)

S1 Table Carbon and nitrogen values of standard material used stable isotopic analysis. Accepted and mean measured (±standard deviation) values of the standard material used in the stable isotopic analysis performed in this study, as well as, the mean minimum and maximum values obtain in each run. The "n" refers to the number of samples of standards materials used.
(DOCX)

S2 Table Breeding phenology of Cape Verde petrels obtained from the light and saltwater immersion data of geolocators. (DOCX)

S3 Table GPS positions of seven tracked domestic cats from Chã das Caldeiras. (XLSX)

S4 Table GPS positions over land of tracked Cape Verde petrels breeding in Fogo island. (XLSX)

S1 Fig **Examples of Cape Verde petrels brood patches representative of the score used in this article:** (A) score 0 –no brood patch (i.e., no evidence of defeathering); (B) score 1 –loss of some down feathers around the edges; (C) score 2 –fully developed brood patch, that in this species occurred when almost all the down feathers fall, but there is still a thin anterior-posterior line of down feathers in the middle of the brood patch; (D) score 3 –similar to score 2 but there are already sheaths of new down feathers appearing; (E) score 4—most of the brood patch area is covered with down feathers that begin to break out of sheaths.
(DOCX)
