# Supplementary material for: Population size, breeding biology and on-land threats of Cape Verde petrel (Pterodroma feae) in Fogo Island, Cape Verde
Source: PLoS One. 2017 Apr 3;12(4):e0174803. doi: 10.1371/journal.pone.0174803 (PMC5378397; doi:10.1371/journal.pone.0174803)
Supplement: S1 Appendix — (DOCX) [file pone.0174803.s001.docx]

## Appendix S1 - Description of molecular sexing methodology

Cape Verde petrel, as many procellariiform species, is monomorphic in plumage coloration and has no obvious sexual dimorphism in size, which makes sex determination in the field extremely difficult. Hence, for molecular sexing of birds, DNA was extracted from ethanol-preserved whole blood using Real Pure genomic DNA extraction kit (Durviz, Spain) and following the manufacturer instructions. The polymerase chain reactions (PCRs) were performed according to the method of [1] and using the following primers: 2550F (5^’^-GTTACTGATTCGTCTACGAGA-3^’^) and 2718R (5^’^-ATTGAAATGATCCAGTGCTTG-3^’^). The PCR reactions were carried out in a total volume of 10 µl containing 1 µl 10x reaction buffer (Bioline), 2 mM MgCl_2_, 0.2 mM of each dNTP, 1 µM of each primer, 0.5 U BioTaq DNA polymerase (Bioline) and about 50 ng of genomic DNA. Amplification products were separated by electrophoresis in a 2% agarose gel stained with RedSafe (Intron Biotechnology) and visualized under ultraviolet light. Sex determination was based on the detection of two homologous sex-linked genes, CHD1-Z, found in both sexes, and CHD1-W, which is female-specific. Therefore, sex was determined as one band in males and two bands in females. This method has been successfully used to identify the sex in a wide variety of bird species, including procellariiformes [1,2].

# References

1. Fridolfsson A-K, Ellegren H. A simple and universal method for molecular sexing of non-ratite birds. Journal of Avian Biology. 1999. pp. 116–121.

2. Fridolfsson A-K, Ellegren H. Molecular evolution of the avian CHD1 genes on the Z and W sex chromosomes. Genetics. 2000;155: 1903–1912.
