# Supplementary material for: Population size, breeding biology and on-land threats of Cape Verde petrel (Pterodroma feae) in Fogo Island, Cape Verde
Source: PLoS One. 2017 Apr 3;12(4):e0174803. doi: 10.1371/journal.pone.0174803 (PMC5378397; doi:10.1371/journal.pone.0174803)
Supplement: S2 Appendix — (DOCX) [file pone.0174803.s002.docx]

## Appendix S2– Description of POPAN Jolly Seber modelling and model selection

Main assumptions of the POPAN Jolly-Seber model are as follows: (1) marks are not lost and can be read correctly; (2) sampling is instantaneous and animals are released immediately after sampling; (3) the study area remains constant; (4) all animals (marked and unmarked) have equal survival probability between each pair of sampling occasions and; (5) all animals (marked and unmarked) have equal capture probability between each pair of sampling occasions [1]. The three first assumptions were met in this study, as rings used were made of stainless steel (which is appropriate to long lived seabirds that spend a lot of time in salt water); birds were released after 15 minutes sampling scheme and the study area remained constant (mist nets were deployed always in the same place). To check if the two last assumptions were met, we tested the goodness-of-fit (GOF) of a fully time-dependent Cormack-Jolly-Seber model using the program U-Care version 2.3.2 [2]. This program allows to performed several tests to those assumption, especially TEST2 and TEST3. TEST2 tests the assumption of equal catchability between marked individuals, which may not be met in case, for example, of existing some trap-dependence. TEST3 tests the assumption that all marked individuals alive at (i) have the same probability of surviving to (i+1). This assumption may not be met if transients animals occur in our study, i.e. animals that leave the study area after their first capture and are not recapture again and thus having a subsequent local survival probability equal to 0 [3].

The POPAN method estimates four demographic parameters: apparent survival probability (ϕ), capture probability (*p*), probability of entrance in the population (*b*), and super-population size (N). The super-population size is defined as the total number of individuals forming part of the population during the study period [4]. The apparent survival, recapture and entry probabilities can be time-dependent or constant through time, being represented by the subscripts "t" and "." respectively. For the fully dependent-model with t occasions, there are t − 1 ϕ estimates, t × *p* estimates, t − 1 *b* estimates, and 1 N estimate. However, there are some confounded parameters (*b*_0_p_1_ and ϕ_(k-1)_p_k),_ that cannot be estimated independently but only as products so the fully time-dependent model has 3k-3 estimable parameters. Regarding to the link functions, we used MLogit link function to estimate *b* parameters, because this link function constraints the sum of the *b* parameters to be 1. To estimate N we used log link function, as super population it is not a value between zero and one; and we used sin link function to estimate ϕ and *p* parameters to avoid extrinsic non-identifiable parameters [5].

We selected the best model using AICc (Akaike Information Criterion corrected for small sample sizes, [6]. AICc is a criterion used to select the most parsimonious model, i.e., the model which best explains the variation in the data while using the fewest parameters. When comparing the AICc values between models, if the difference between them (∆AICc) is lower than 2, it is reasonable to considered that both models have approximately equal weight in the data [7]. However if 4 <∆AICc < 7 there is a considerable support for a real difference between the models and in the cases of ∆AICc > 10 the model with higher AICc failed to explain substantial variation in the data and might be omitted from further consideration [7]. From the AICc values of each model an Akaike weight (AICc weight) is also computed. These AICc weight are then normalized to sum 1, i.e., the weight of each model is divided by the sum of the weights of all models considered. AICc weight values reflects the likelihood of the model given the data, if the most parsimonious model has AICc weight of ≥90, it means that this model comprises ≥90% of support in the data and inferences can be made considering only this model [5]. But if none of the models shows AICc weight of ≥90, then model averaging should be perform to take into account the model selection uncertainty [7]. Model averaging is calculated considering the relative importance of each fitted model using the normalized AICc weights [7].

# References

1. Schwarz CJ, Arnason AN. Jolly-Seber models in MARK. In: Cooch E, White G, editors. Program Mark: a gentle introduction 13th edition. 2014. pp. 1–52.

2. Choquet R, Reboulet A-M, Lebreton J-D, Gimenez O, Pradel R. U-CARE 2.2 user’s manual. Montpellier: Centre d’Ecologie Fonctionnéllé & Evolutive; 2005.

3. Pradel R, Hines JE, Lebreton J-D, Nichols JD. Capture-recapture survival models taking account of transients. Biometrics. 1997;53: 60–72. doi:10.2307/2533097

4. Crosbie SF, Manly BFJ. Parsimonious modelling of capture-mark-recapture studies. Biometrics. 1985;41: 385–398. Available: http://www.jstor.org/stable/2530864

5. Cooch EG, White GC. Program MARK, A gentle introduction 13^th^ edition [Internet]. 2014. pp. 1–414. Available: papers2://publication/uuid/60AD4742-AFB2-43CE-82E5-08D1F9EDC916

6. White GC, Burnham KP. Program MARK: survival estimation from populations of marked animals. Bird Study. 1999;46: S120–S139.

7. Burnham KP, Anderson DR. Model selection and multimodel inference: a practical information-theoretic approach [Internet]. 2nd ed. New York: Springer; 2002. Available: http://linkinghub.elsevier.com/retrieve/pii/S0304380003004526
