# Supplementary material for: Population size, breeding biology and on-land threats of Cape Verde petrel (Pterodroma feae) in Fogo Island, Cape Verde
Source: PLoS One. 2017 Apr 3;12(4):e0174803. doi: 10.1371/journal.pone.0174803 (PMC5378397; doi:10.1371/journal.pone.0174803)
Supplement: S3 Appendix — (DOCX) [file pone.0174803.s003.docx]

## Appendix S3 – Description of the stable isotope analysis of fur samples

All fur samples were cut from the dorsal of the animal, cleaned with 4 rinses of chloroform:methanol 2:1 and dried in an oven at 60ºC for 48h. Samples were ground to powder with scissors and a subsample of 0.275 mg (0.26-0.3mg) was placed into a 0.03 ml tin capsule and dry-preserved until analysis. The preparation and analyses of fur sampled followed the "principle of identical treatment" [1]. Isotope ratios are expressed conventionally as *δ* values in part per thousand (‰) according to the following equation:

*δ*X = [(Rsample/Rstandard) – 1]

where X (‰) is ^13^C or ^15^N and R are the corresponding ratio ^13^C/^12^C or ^15^N/^14^N related to the standard values. Rstandard for ^13^C is Vienna Pee Dee Belemnite (VPDB) and for ^15^N is atmospheric nitrogen (AIR). The isotopic ratio mass spectrometry facility at the Serveis Científico-Tècnics of the University of Barcelona (Spain) applied international standards (IAEA CH_7_, IAEA CH_6_, IAEA600 and USGS 40 for C and IAEA N1, IAEA N_2_, IAEA 600, IAEA NO_3_ and USGS 40 for N) and inserted two standard material samples every 12 fur samples to calibrate the system and compensate for any drift over time. Replicate assays of standard material samples indicated standard deviation of ± 0.2 for carbon and nitrogen (see Table S1 in supporting information)

# References

1. Bond AL, Hobson KA. Reporting stable-isotope ratios in ecology: recommended terminology, guidelines and best practices. Waterbirds. 2012;35: 324–331.
