# Supplementary material for: Population size, breeding biology and on-land threats of Cape Verde petrel (Pterodroma feae) in Fogo Island, Cape Verde
Source: PLoS One. 2017 Apr 3;12(4):e0174803. doi: 10.1371/journal.pone.0174803 (PMC5378397; doi:10.1371/journal.pone.0174803)
Supplement: S1 Table — Accepted and mean measured (±standard deviation) values of the standard material used in the stable isotopic analysis performed in this study, as well as, the mean minimum and maximum values obtain in each run. The "n" refers to the number of samples of standards materials used. (DOCX) [file pone.0174803.s004.docx]

**Table S1 - Accepted and mean measured (±standard deviation) values of the standard material used in the stable isotopic analysis performed in this study.**

| Standard material name | *δ*^15^N_Air_ (‰) | | | | *δ*^13^C_VPDB_ (‰) | | | | References |
| --- | --- | --- | --- | --- | --- | --- | --- | --- | --- |
|  | Accepted value ± SD | Measured values | | | Accepted value ± SD | Mean measured values | | |  |
|  |  | n | Mean of all runs ± SD | Minimum - maximum mean within runs |  | n | Mean of all runs ± SD | Minimum - maximum mean within runs |  |
| IAEA CH6 |  | 2 |  |  | –10.449±0.033 | 2 | –10.7±0.1 | –10.6 to –10.7 | [1] |
| IAEA CH7 |  | 2 |  |  | –32.151±0.050 | 2 | –32.2±0.1 | –32.1 to –32.3 | [1] |
| IAEA 600 | +1.0±0.2 | 3 | +0.93±0.2 | 0.8 to 1.1 | –27.771±0.043 | 3 | –27.6±0.3 | –27.9 to –26.9 | [1] |
| USGS 40 | –4.52±0.06 | 2 | –4.51±0.1 | –4.4 to -4.6 | –26.24±0.07 | 2 | –26.5±0.1 | –26.4 to –26.6 | [2] |
| IAEA N1 | +0.43±0.07 | 4 | +0.31±0.2 | 0.04 to 0.46 |  |  |  |  | [3] |
| IAEA N2 | +20.41±0.12 | 2 | +19.42±0.1 | 19.4 to 19.6 |  |  |  |  | [3] |
| IAEA NO3 | +4.72±0.13 | 2 | +4.28±0.07 | 4.2 to 4.3 |  |  |  |  | [3] |

Legend: Accepted and mean measured (±standard deviation) values of the standard material used in the stable isotopic analysis performed in this study, as well as, the mean minimum and maximum values obtain in each run. The "n" refers to the number of samples of standards materials used.

# References

1. Coplen TB, Brand WA, Gehre M, Gro M, Meijer HAJ, Toman B, et al. New guidelines for δ^13^C measurements. Anal Chem. 2006;78: 2439–2441.

2. Qi H, Coplen TB, Gelimann H, Brand WA, Böhlke JK. Two new organic reference materials for δ^13^C and δ^15^N measurements and new value for the δ^13^C of NBS 22 oil. Rapid Commun Mass Spectrom. 2003;17: 2483–2487.

3. Böhlke JK, Coplen TB. Interlaboratory comparison of reference materials for nitrogen-isotope-ratio measurements. Reference and intercomparison materials for stable isotopes of light elements. Viena: Proceedings of a consultants meeting - IAEA; 1993. pp. 51–66.
