# Supplementary material for: Population size, breeding biology and on-land threats of Cape Verde petrel (Pterodroma feae) in Fogo Island, Cape Verde
Source: PLoS One. 2017 Apr 3;12(4):e0174803. doi: 10.1371/journal.pone.0174803 (PMC5378397; doi:10.1371/journal.pone.0174803)
Supplement: S2 Table — (DOCX) [file pone.0174803.s005.docx]

**Table S2 – Breeding phenology of Cape Verde petrels obtained from the light and saltwater immersion data of geolocators.**

| **Ring** | **Breeding season** | **Sex** | **1^st^ night at nest (nº hours on dry)** | **1^st^ day at nest** | **1^st^ incubation bout** | **Duration 1^st^ incubation (days)** | **2^nd^ incubation bout** | **Duration 2^nd^ incubation (days)** |
| --- | --- | --- | --- | --- | --- | --- | --- | --- |
| **5500481** | 2011/2012 | Female | 09/10/2011 (5.67h) | 12/11/2011 | 29/01/2012 | 15 | 25/02/2012 | 12 |
| **5500481** | 2012/2013 | Female | 30/09/2012 (5.33h) | 10/11/2012 | 28/01/2013 | 14 | 26/02/2013 | 7 |
| **5500481** | 2013/2014 | Female | 03/10/2013 (5.17h) | 12/11/2013 | 31/01/2014 | 10 | - | - |
| **5500347** | 2011/2012 | Female | - | - | - | - | 03/03/2012 | 13 |
| **5500347** | 2012/2013 | Female | 23/09/2012 (5.22h) | 21/11/2012 | 04/02/2012 | 22 | 09/03/2012 | 3 |
| **5500347** | 2013/2014 | Female | 27/09/2013 (4.63h) | 15/11/2013 | - | - | - | - |
| **5500071** | 2011/2012 | Female | - | - | - | - | 29/02/2012 | 11 |
| **5500071** | 2012/2013 | Female | 19/09/2012 (5.33h) | 18/11/2012 | 05/02/2013 | - | - | - |
| **5500083** | 2011/2012 | Male | - | - | - | - | 13/02/2012 | 12 |
| **5500083** | 2012/2013 | Male | 03/09/2012 (4.83) | 02/11/2012 | 05/01/2013 | 14 | 03/02/2013 | 15 |
| **Mean (±SD)** |  |  | 24 Sep (±12) | 12 Nov (±6) | 27 Jan (±11) | 15 (±4) | 22 Feb (±12) | 10 (±4) |
