# Supplementary material for: Population size, breeding biology and on-land threats of Cape Verde petrel (Pterodroma feae) in Fogo Island, Cape Verde
Source: PLoS One. 2017 Apr 3;12(4):e0174803. doi: 10.1371/journal.pone.0174803 (PMC5378397; doi:10.1371/journal.pone.0174803)

**Fig S1 – Examples of Fea’s petrels brood patches representative of the score used in this article:** (A) score 0 – no brood patch (i.e., no evidence of defeathering); (B) score 1 – loss of some down feathers around the edges; (C) score 2 – fully developed brood patch, that in this species occurred when almost all the down feathers fall, but there is still a thin anterior-posterior line of down feathers in the middle of the brood patch; (D) score 3 – similar to score 2 but there are already sheaths of new down feathers appearing; (E) score 4 - most of the brood patch area is covered with down feathers that begin to break out of sheaths.


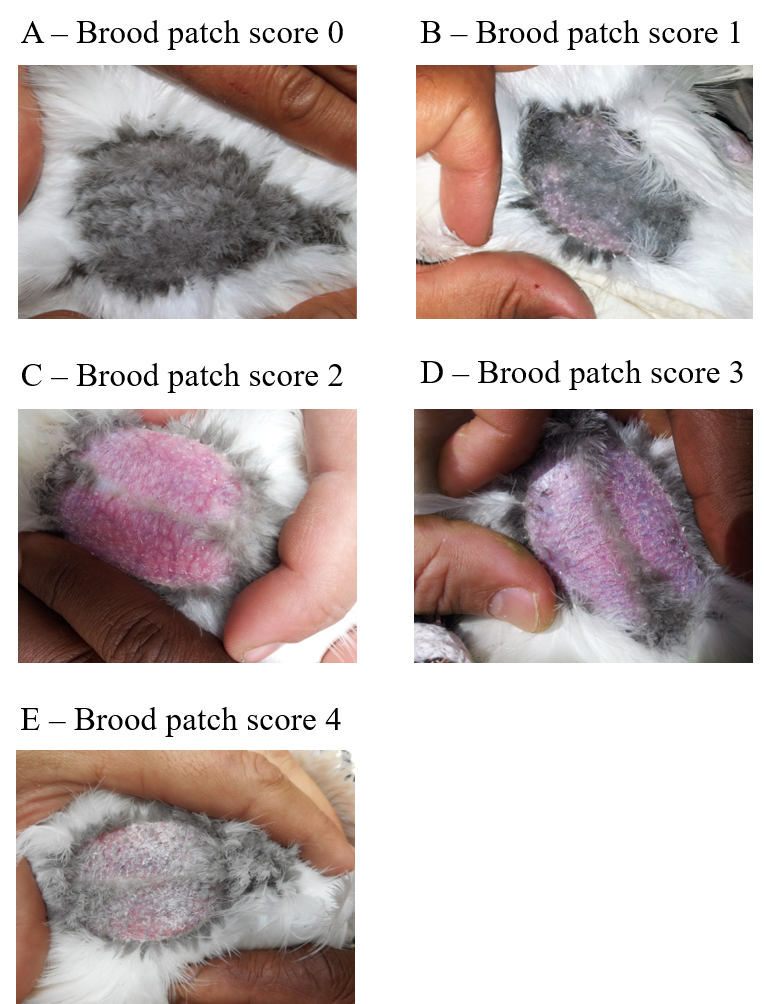

Supplement: S1 Fig — Examples of Cape Verde petrels brood patches representative of the score used in this article: (A) score 0 –no brood patch (i.e., no evidence of defeathering); (B) score 1 –loss of some down feathers around the edges; (C) score 2 –fully developed brood patch, that in this species occurred when almost all the down feathers fall, but there is still a thin anterior-posterior line of down feathers in the middle of the brood patch; (D) score 3 –similar to score 2 but there are already sheaths of new down feathers appearing; (E) score 4—most of the brood patch area is covered with down feathers that begin to break out of sheaths. (DOCX) [file pone.0174803.s008.docx]
